# Genotypic Variation in the Root and Shoot Metabolite Profiles of Wheat (*Triticum aestivum* L.) Indicate Sustained, Preferential Carbon Allocation as a Potential Mechanism in Phosphorus Efficiency

**DOI:** 10.3389/fpls.2019.00995

**Published:** 2019-08-06

**Authors:** Van Lam Nguyen, Lachlan Palmer, Ute Roessner, James Stangoulis

**Affiliations:** ^1^College of Science and Engineering, Flinders University, Bedford Park, SA, Australia; ^2^Department of Biochemistry and Food Biotechnology, Vietnam National University of Agriculture, Hanoi, Vietnam; ^3^School of Biosciences, The University of Melbourne, Melbourne, VIC, Australia

**Keywords:** metabolomics, phosphorus use efficiency, wheat, root exudates, mechanism

## Abstract

Changes in the levels of plant metabolites in response to nutrient deficiency is indicative of how plants utilize scarce resources. In this study, changes in the metabolite profile of roots and shoots of wheat genotypes differing in phosphorus use efficiency (PUE) was investigated. Under low P supply and at 28 days after sowing (DAS), the wheat breeding line, RAC875 (P efficient) produced 42% more shoot biomass than the wheat variety, and Wyalkatchem (P inefficient). Significant changes in the metabolite profile in leaves and roots were observed under low P supply and significant genotypic variation was evident. Under low P supply, an increase in raffinose and 1-kestose was evident in roots of both wheat genotypes, with RAC875 accumulating more when compared to Wyalkatchem. There was no significant increase in raffinose and 1-kestose in leaves when plants were grown under P deficiency. P deficiency had no significant impact on the levels of sucrose, maltose, glucose and fructose in both genotypes, and while phosphorylated sugars (glucose-6-P and fructose-6-P) remained unchanged in RAC875, in Wyalkatchem, glucose-6-P significantly decreased in roots, and fructose-6-P significantly decreased in both leaves and roots. Glycerol-3-P decreased twofold in roots of both wheat genotypes in response to low P. In roots, RAC875 exhibited significantly lower levels of fumarate, malate, maleate and itaconate than Wyalkatchem, while low P enhanced organic acid exudation in RAC875 but not in Wyalkatchem. RAC875 showed greater accumulation of aspartate, glutamine and β-alanine in leaves than Wyalkatchem under low P supply. Greater accumulation of raffinose and 1-kestose in roots and aspartate, glutamine and β-alanine in leaves appears to be associated with enhanced PUE in RAC875. Glucose-6-P and fructose-6-P are important for glycolysis, thus maintaining these metabolites would enable RAC875 to maintain carbohydrate metabolism and shoot biomass under P deficiency. The work presented here provides evidence that differences in metabolite profiles can be observed between wheat varieties that differ in PUE and key metabolic pathways are maintained in the efficient genotype to ensure carbon supply under P deficiency.

## Introduction

Phosphorus (P) is a vital macronutrient for plant growth and development and plays a variety of cellular functions including structural roles (i.e., nucleic acids and phospholipids), energy transfer (i.e., ATP) and phosphorylated intermediates (i.e., glucose-6-P, fructose-6-P), and therefore it is involved in the regulation of metabolic pathways (Huang et al., 2008; Muller et al., 2015). However, available soil P is low in many agricultural soils and this reduces crop growth and productivity (Lynch, 2011). Besides, P fertilizer resources are limited and they are predicted to be depleted by 2050 (Vance et al., 2003), therefore the development of plants with improved phosphorus use efficiency (PUE) is critical to cope with this issue.

Phosphorus is taken up by plant roots in the form of inorganic phosphorus (P_i_) (Schachtman et al., 1998). The concentration of P_i_ in the soil is generally much lower than the P_i_ concentration in root cells, thus plants use active transport systems to acquire P from the soil solution. This process is carried out by high-affinity P_i_/H^+^ symporters that are controlled by two major gene families (*Pht1* and *Pht2*) (Cui et al., 2011). Once in the roots, P_i_ is partly used to synthesize P-containing substances such as ATP or phospholipids, or can be stored in the vacuole, while its main fraction is transported into xylem vessels and allocated to the stem, leaves, flowers, and seeds (Młodzińska and Zboińska, 2016). The high-affinity P_i_/H^+^ symporters are not only involved in P uptake but also in P translocation throughout the plant. These transporters are predicted to be membrane-spanning proteins with 12 domains (Rausch and Bucher, 2002).

Plants respond to low P through two main approaches, including the modification of root system architecture (RSA) and alterations of metabolic processes (Jones et al., 2015; Wang et al., 2015). Changes in RSA in response to P deficiency is often related to P uptake, while modifications of metabolic processes is involved in P utilization (Niu et al., 2013). Plants modify metabolic pathways to enhance internal P efficiency (Vance et al., 2003). For example, plants can replace phospholipids in membranes by sulfolipids and galactolipids, thus reducing P requirements, and enabling the plant to utilize P more efficiently (Veneklaas et al., 2012). Also, under P deficiency, instead of using P_i_, plants can activate metabolic bypass enzymes that depend on pyrophosphate (PP_i_) (Plaxton and Tran, 2011). There are PP_i_-dependent glycolytic enzymes that allow plants to maintain the carbon flux under P starvation (Plaxton and Podesta, 2006). This activation of alternative metabolic pathways could result in changes in tissue metabolites and this has been reported under P deficiency where studies have shown that plants accumulate sugars under P starvation. For example, di- and trisaccharides (sucrose, maltose, and raffinose) increased in barley (Huang et al., 2008) and maize (Ganie et al., 2015) under P deprivation. Accumulation of sugars was also observed in bean roots (Rychter and Randall, 1994) and different cucumber tissues (Ciereszko et al., 2002) under low P. Thus, the accumulation of sugars under low P appears to be a P efficiency mechanism. However, a study in lupin was able to show that fructose, glucose and sucrose in shoots declined after 14 days of P deficiency and after 22 days of P deficiency, no effect on sugar levels in shoots and roots was found (Muller et al., 2015).

A decrease in phosphorylated sugars could be a plant adaptive mechanism that helps plants to use P more efficiently under low P supply. Low P resulted in reductions of glucose-6-P, fructose-6-P and inositol-P in barley (Huang et al., 2008), maize (Ganie et al., 2015), and lupin (Muller et al., 2015). A decrease in phosphorylated sugars was also observed in other plants (Rychter and Randall, 1994; Warren, 2011), so maintenance of phosphorylated sugars under low P could in theory, and help in maintaining carbohydrate metabolism. Plants also respond to low P through alterations in amino acid and organic acid levels. For example, glutamine and asparagine increased, while organic acids (i.e., α-ketoglutarate, succinate, fumarate, and malate) also increased (Huang et al., 2008). The field of metabolomics is rapidly emerging as an effective tool to elucidate key metabolic pathways leading to stress tolerance. Jewett et al. (2006) claim that metabolites can “act as spoken language, broadcasting signals from the genetic architecture and the environment,” and one can see how this tool has the ability to give a real-time representation of a plants physiological state. Metabolomics has also had its challenges and these are reviewed in Roessner and Bowne (2009). The concept of one type of analysis fitting all is simply not possible and this then requires proficiency in the use of various analytical platforms such as GC-MS and LC-MS. We are fortunate that sophisticated statistical and multi-variant data analysis tools are freely available and this helps to discriminate between noise and real sample-related information. Perhaps the most complex challenge is to put any results gained into a biological context.

Although metabolites have been profiled in wheat under abiotic stresses, such as drought and salt stress (Bowne et al., 2012), there is little evidence of metabolite profiles in wheat grown under P stress. This study aimed to investigate mechanisms of PUE through profiling metabolites and root exudates of two wheat genotypes, RAC875 (P efficient), and Wyalkatchem (P inefficient) under P starvation. Identifying P efficiency mechanisms provides further sites for genetic manipulation and can lead to the development of P-efficient wheat varieties.

## Materials and Methods

### Plant Materials

Seed of the wheat (*Triticum aestivum* L.) genotypes, RAC875 and Wyalkatchem were sourced from Dr Glenn McDonald at the University of Adelaide. Prior to planting, seed was soaked for 5 min in sodium hypochlorite, rinsed in high purity water (>18.2 MΩ cm^–1^ resistivity) and pre-germinated for 3 days in petri-dishes lined with filter paper wet with high purity water (>18.2 MΩ cm^–1^ resistivity). Seedlings were planted in washed sand with basal nutrients added as outlined in Table 1. We were mindful of the effects of N on P (Ziadi et al., 2008) so optimal N was supplied. The basal nutrient solution was added to dry sand weighed out into plastic bags and thoroughly mixed prior to planting. Plants were treated as outlined in the following experimental set-ups.

**TABLE 1:** Shoot DM, root DM, root to shoot ratio, and rhizosheath size of two wheat genotypes grown under the growth room conditions at 28 DAS.

**P supply (mg P kg^–1^)**	**Genotype**	**Shoot DM (g plant^–1^)**	**Root DM (g plant^–1^)**	**Root to shoot ratio (g g^–1^)**
10	RAC875	0.44 ± 0.01^a**^	0.33 ± 0.02^a^	0.76 ± 0.05^a^
	Wyalkatchem	0.31 ± 0.03^b^	0.28 ± 0.02^a^	0.90 ± 0.03^a^
Average		0.38 ± 0.03	0.31 ± 0.02	0.83 ± 0.04
30	RAC875	0.52 ± 0.01^a^	0.37 ± 0.05^a^	0.64 ± 0.01^a**^
	Wyalkatchem	0.43 ± 0.04^a^	0.33 ± 0.02^a^	0.77 ± 0.03^b^
Average		0.47 ± 0.02	0.35 ± 0.03	0.71 ± 0.03
*P values*				
Genotype (G)		*P* = 0.001	*P* = 0.136	*P* = 0.002
P treatment (P)		*P* = 0.004	*P* = 0.181	*P* = 0.006
G × P		*P* = 0.472	*P* = 0.872	*P* = 0.948

*Results represent means ± standard errors of four biological replicates. *P* values are from two-way ANOVA analysis. Different letters show significant differences between genotypes at each P supply and ^∗∗^ following letters: significant at *P* < 0.01, respectively; comparisons were performed by independent Student’s *t*-test.*

#### Experimental Set-Up 1: Effect of P Supply on Leaf P Status and Metabolite Profiles of Genotypes With Contrasting PUE

Plants were grown in square-shaped 18.0 cm high × 8.5 cm wide pots lined with plastic bags holding 1.1 kg of sandy soil. The properties of sandy soil were described in Supplementary Table S1. Two P levels were used; 10 and 30 mg P kg^–1^ soil. Three seedlings were planted into each pot with four biological replicates and grown in a growth room. Growth room conditions were 13/11 h light/dark at 20/10°C with a minimum of 660 μmol m^–2^ s^–1^ photosynthetic photon flux density at the leaf surface. Plants were watered three times a week to 10% of soil weight. One plant was thinned from each pot at 7 and 15 DAS and the remaining plant harvested at 28 DAS. The two youngest leaves from the primary tillers and older leaves were sectioned and stems were detached from the roots at the crown level. All leaves and stems were quickly rinsed with high purity water (>18.2 MΩ cm^–1^ resistivity), transferred to 50 mL tubes, snap frozen in liquid N and stored at −80°C. The roots were washed with high purity water (>18.2 MΩ cm^–1^ resistivity), dried using paper towel, weighed, transferred to 50 mL tubes, snap frozen in liquid N, and stored at −80°C. Tissue samples were freeze-dried for 48 h and weighed prior to metabolite profiling.

To test the level of deficiency witnessed by the plants at the time of sampling, young leaf tissue was analyzed by ICP-MS (Agilent Technologies, Model 7500cx) at Flinders University using the closed-tube digestion method developed by Wheal et al. (2011).

#### Experimental Set-Up 2: Effect of P Supply on Root Exudate Composition of Genotypes With Contrasting PUE

Plants were grown in 4.2 kg of sandy soil in pots 18.5 cm deep × 17.5 cm top diameter lined with plastic bags, at three P levels; 5, 10, and 30 mg P kg^–1^ soil. Before seedlings were planted, a 0.15 μm pore size, 10 cm porous length Rhizon (Rhizosphere Research Product, Netherlands) was diagonally (∼45°) inserted into each pot allowing approximately 2 cm clearance from the bottom of the pot. One seedling was planted in each pot with four biological replicates. Plants were grown under greenhouse conditions for the first 34 DAS, then transferred to a growth room. Growth room conditions were 13/11 h light/dark at 20/10°C with a minimum of 660 μmol m^–2^ s^–1^ photosynthetic photon flux density at the leaf surface. Plants were watered three times a week to 10% of soil weight.

Unlike Experimental Set-up 1, plants were left longer in pots before exudates were collected and this was to encourage a larger root system so that sufficient exudate could be collected. At 41 DAS, plants were watered to 9% of soil weight and after 1 h, 15 ml of soil solution was extracted from the rhizon using a 20 mL syringe over 30 min. Samples were transferred to a labeled 15 mL tube and stored at −80°C. Soil solution samples were then freeze dried and reconstituted with 2 mL of high purity water (>18.2 MΩ cm^–1^ resistivity). The organic acids in the soil solution were extracted with THF as detailed in section “Organic Acid Extraction Optimization” and analyzed using High Pressure Ion Chromatography (HPIC) as described in section “HPIC conditions.”

### Metabolite Profiling

#### Metabolite Extraction and TMS Derivatization

Metabolites were extracted as described by Hill et al. (2013), with modifications. In brief, 15 mg of freeze-dried shoot or root material were transferred to Cryo-mill tubes and accurate weights were recorded. Methanol (MeOH, 600 μL) containing the following internal standards, ^13^C_6_-Sorbitol (0.02 mg mL^–1^) and ^13^C_5_-^15^N-Valine (0.02 mg mL^–1^), was added to the sample tubes. The samples were homogenized using a Cryo-mill [Berting Technologies; program #2 6800-3 × 30 × 30 at −10°C)] and then incubated in a Thermomixer at 30°C with a mixing speed of 900 rpm for 15 min, followed by 5 min of centrifugation at 15,000 rpm (21,200 × *g*). The MeOH supernatant was transferred into a 1.5 mL Eppendorf tube and set aside. Water (600 μL) was added to the remaining sample pellet and vortexed before being centrifuged for 10 min at 15,000 rpm (21,200 × *g*). The supernatant was removed and combined with the MeOH supernatant. Aliquots of 50 μL were transferred to a clean glass insert in Eppendorf tubes and dried *in vacuo* using a Rotational Vacuum Concentrator (RVC 2-33 CD plus, John Morris Scientific, Pty Ltd., Melbourne, VIC, Australia) at ambient temperature.

Dried samples (*n* = 4 biological replications) were prepared by the addition of 20 μL of Methoxyamine Hydrochloride (30 mg mL^–1^ in Pyridine) followed by shaking at 37°C for 2 h. The sample was then derivatized with 20 μL of *N*,*O*-*bis* (Trimethylsilyl)trifluoroacetamide with Trimethylchlorosilane (BSTFA with 1% TMCS, Thermo Fisher Scientific) for 30 min at 37°C. The sample was then left for 1 h before 1 μL was injected onto the GC column using a hot needle technique. Splitless and split (1:20) injections were done for each sample.

#### Metabolite Analysis by GC-MS

The GC-MS system used comprised of a Gerstel 2.5.2 autosampler, a 7890A Agilent gas chromatograph and a 5975C Agilent quadrupole mass spectrometer (Agilent, Santa Clara, CA, United States). The mass spectrometer was tuned according to the manufacturer’s recommendations using tris-(perfluorobutyl)-amine (CF43).

Gas chromatography was performed on a 30 m Agilent J & W VF-5MS column with 0.25 μm film thickness and 0.25 mm internal diameter with a 10 m Integra guard column. The injection temperature (Inlet) was set at 250°C, the MS transfer line at 280°C, the ion source adjusted to 230°C, and the quadrupole at 150°C. He was used as the carrier gas at a flow rate of 1 mL min^–1^.

The analysis of TMS-derivatized samples was performed under the following temperature program; start at injection 70°C, a hold for 1 min, followed by a 7°C min^–1^ oven temperature ramp to 325°C and a final 6 min heating at 325°C. Mass spectra were recorded at 2.66 scans.s^–1^ with an 50–600 *m/z* scanning range.

#### Data Processing

Data were processed using the Agilent MassHunter Quantitative Analysis version B.07.00 software. Mass spectra of eluting TMS-derivatized compounds were identified using the commercial mass spectra library NIST^1^, the public domain mass spectra library of Max-Planck-Institute for Plant Physiology, Golm, Germany^2^, and the *in-house* Metabolomics Australia mass spectral library. Resulting relative response ratios normalized per mg dry weight for each analyzed metabolite were prepared as described by Roessner et al. (2001).

### Organic Acid Analysis From Root Exudates

#### Organic Acid Extraction Optimization

Soil solution was simply extracted using rhizons, however, organic acids were poorly separated through the Dionex system (Ion Chromatography) due to the effect of anions from the fertilizer solution, with NO_3_^–1^ and SO_4_^–2^ being particularly problematic. Therefore, organic acids needed to be extracted from soil solution before analysis by HPIC. The initial organic acid extraction protocol used in this work was derived from the work presented by Ding et al. (2006). Some of the modifications are based on the evidence provided by the work presented by Wittmann et al. (2008). Two solvents, ethyl acetate (EtAc) and Tetrahydrofuran (THF) were tested for their ability to extract organic acids from the collected soil solution.

A test solution containing 25 μM of eight organic acids (maleic, malic, citric, succinic, oxalic, fumaric, tartaric, and malonic), 2 mM NO_3_^–^ [from Ca(NO_3_)_2_] and 1 mM SO_4_^2–^ (from K_2_SO_4_) was prepared using high purity water (>18.2 MΩ cm^–1^ resistivity). To a 2 mL aliquot of the test solution, 25 μL of 6M HCl was added and then saturated with NaCl (0.7 g). The solution was vortexed briefly and 2 mL of solvent was added, either EtAc or THF, vortexed for 30 s, and then shaken on a rotatory mixer for 15 min. The extraction solution was centrifuged at 800 × *g* for 5 min. The solvent phase was collected, and the extraction was repeated a second time on the aqueous layer. The solvent from the two extractions was combined and evaporated under the N_2_ flow using REACTI-VAP II (Thermo Fisher Scientific). The test samples were reconstituted in 1 mL of Milli-Q water immediately prior to analysis by high pressure ion chromatography (HPIC).

A second test solution was prepared with 25 μM of eight organic acids (maleic, malic, citric, succinic, oxalic, fumaric, tartaric, and malonic) in a bulked soil solution collected from multiple pots and recovery was explored using only THF as detailed above.

All recovery tests were done in triplicate.

#### HPIC Conditions

The HPIC method is based on the method outlined in Dionex application note 143 with modifications based on information provided in the IonPac AS11-HC product manual (Thermo Fisher Scientific, 031333-09).

The HPIC was conducted using an ICS-3000 HPLC system (Thermo Fisher Scientific) fitted with a quaternary pump, equipped with an eluent generator (EGC-KOH), an IonPac AG11-HC guard column (2 mm × 50 mm, 052963) coupled to a IonPac AS11-HC analytical column (2 mm × 250 mm, 052961) and suppressed conductivity detection (ASRS 300, 2 mm suppressor) in external water mode (1 mL min^–1^).

For each analysis, 10 μL of sample was injected and separated using a mixture of KOH and methanol as per the separation method outlined in Supplementary Table S2. The flow rate used was 0.38 mL min^–1^ and the column and detection compartments were maintained at 30°C.

### Statistical Analysis

Statistical analyses for shoot DM, root DM, root to shoot ratio and organic acid contents were conducted in IBM SPSS v23. The normality of data was tested using Kolmogorov-Smirnov and Shapiro-Wilk tests (*P* < 0.05). These parameters were analyzed by two-way ANOVA (Genotype × P supply). Mean comparisons between genotypes at each P treatment were performed by independent *t*-test (*P* < 0.05) (Field, 2013). Differences between sample groups for metabolites were validated using the Student’s *t*-test (*p*-value < 0.05).

## Results

### Responses to P of Two Wheat Genotypes at 28 DAS

Shoot P concentration differed significantly (*P* < 0.001) between the treatments indicating a significant difference between the P status of the genotypes tested. At 10 mg P kg^–1^ soil, RAC875 and Wyalkatchem had a P concentration of 0.27 and 0.28%, respectively, while at a P supply of 30 mg P kg^–1^ soil, the shoot P concentration was 0.54 and 0.67% respectively. The genotype (G) × P treatment was significant (*P* < 0.05). Given the concentrations in shoot tissue at low P supply, this corresponds to a deficiency status according to Reuter and Robinson (1997).

In contrast to leaf tissue P responses, there were no significant G × P supply (P) interactions for shoot DM, root DM and root to shoot ratio. Low P significantly (*P* < 0.01) reduced shoot DM from 0.47 ± 0.02 to 0.38 ± 0.03 g DW plant^–1^, but did not affect root dry matter (DM) (Table 1). In contrast to shoot DM, when grown in low P soils, wheat plants significantly (*P* < 0.01) increased the root:shoot ratio when compared to plants grown at adequate P.

Under low P, shoot DM of RAC875 was 0.44 ± 0.01 g DW plant^–1^ and showed 42% higher (*P* < 0.01) shoot DM than Wyalkatchem, while no significant difference occurred under adequate P, but no significant (*P* = 0.11) variation in root DM was observed between the wheat genotypes (Table 1).

Phosphorus use efficiency was calculated as relative shoot DM between low and adequate P supply, by which RAC875 showed 12.5% greater PUE than Wyalkatchem.

### Changes in Tissue Metabolite Profiles Under P Deficiency in Leaf and Root Tissues

This study used a comparative GC-MS based method to identify metabolic changes under low P compared with adequate P and differences in metabolites between two wheat genotypes with contrasting PUE in response to P starvation. A total of 79 and 84 metabolites were measured in the shoots and roots of the two wheat genotypes, respectively. These metabolites include amino acids and amines, organic acids and sugars and sugar phosphates.

**FIGURE 1:**
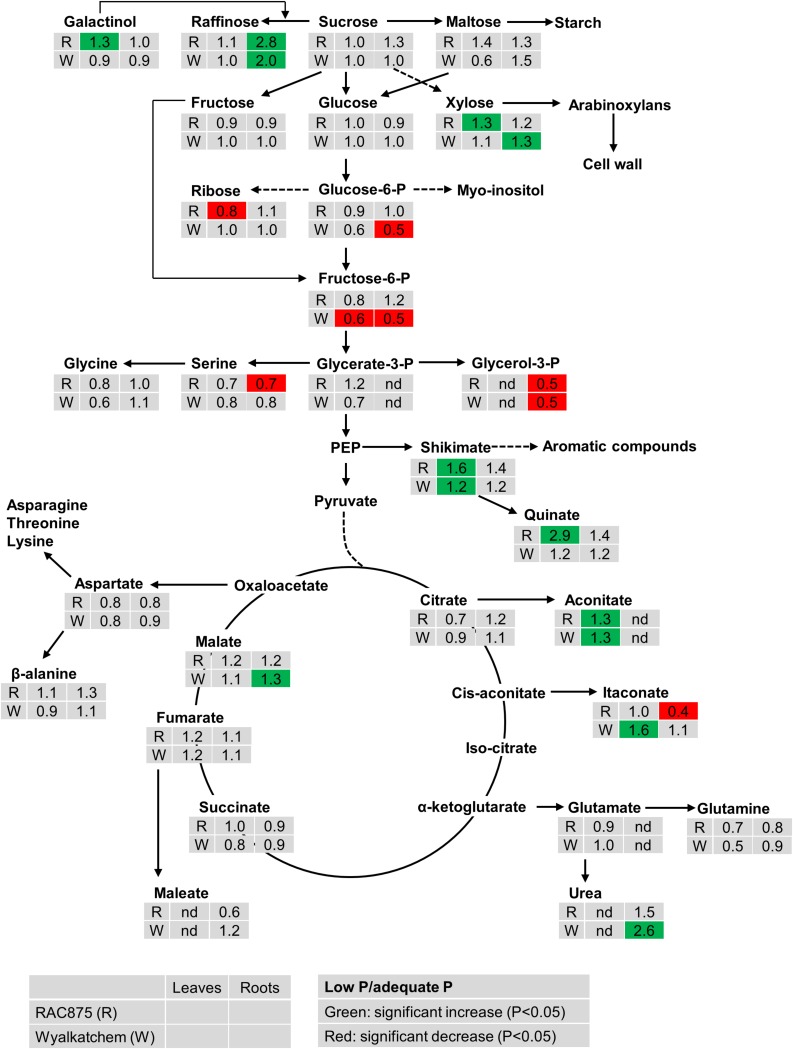
The effect of P supply on the levels of metabolites from shoots and roots of two wheat genotypes RAC875 (the upper row) and Wyalkatchem (the lower row) at 28 days after sowing (DAS). Plants were grown in sandy soils at low (10 mg P kg^–1^ soil) and adequate (30 mg P kg^–1^ soil) P supply. Relative ratios (low P/adequate P) in shoots (the first column) and in roots (the second column) are presented as means of four biological replicates. Significant increases (*P* < 0.05) are indicated in green and significant decreases (*P* < 0.05) are indicated in red; nd: not detectable.

The effect of P supply on metabolite levels in leaves and roots of the two wheat genotypes can be seen in the pathway analysis in Figure 1 and Supplementary Figures S1, S2. From the results, the effects of P supply are clearly more evident in the root than in the leaves. In leaves, low P did not significantly affect levels of sucrose, maltose, raffinose, glucose and fructose in both wheat genotypes, however, xylose significantly increased (1.3-fold) in leaves of RAC875 but not in Wyalkatchem leaf tissue. Ribose significantly decreased (0.8-fold) in leaves of RAC875 but not in Wyalkatchem leaves. Under low P, the concentration of leaf galactinol, a sugar of raffinose oligosaccharides (RFOs), increased in RAC875 (1.3-fold) while a slight decrease was observed in Wyalkatchem. Similar to the effect in leaves, under low P there was no significant change in sucrose, maltose, glucose, and fructose levels within the roots. There was however, a significant increase in raffinose for both RAC875 (2.8-fold) and Wyalkatchem (2.0-fold) under P deficiency, when compared to adequate P. The level of 1-kestose in roots also increased under low P in both wheat genotypes. Low P significantly enhanced arabinose, galactosylglycerol, mannose, and xylose levels in roots of Wyalkatchem but these sugar levels remained unchanged in RAC875, between the P treatments.

Under low P, the levels of the phosphorylated sugar glucose-6-P significantly reduced (0.5-fold) in Wyalkatchem roots while fructose-6-P significantly decreased in both leaves (0.6-fold) and roots (0.5-fold) in Wyalkatchem. Meanwhile, low P had no significant effect on these phosphorylated metabolites in RAC875 (Figure 1). Under low P, glycerol-3-P level decreased 0.5-fold in roots of both RAC875 and Wyalkatchem (Figure 1).

The levels of most organic acids of the tricarboxylic acid (TCA) cycle (citrate, succinate, and fumarate) remained unchanged under low P in both leaves and roots of the two wheat genotypes (Figure 1). However, low P promoted significantly (*P* < 0.05) higher levels of leaf aconitic acid (1.3-fold) in both RAC875 and Wyalkatchem. In leaves, low P increased levels of isocitric acid (1.2-fold), and malonic acid (1.6-fold) in RAC875 but no change in these organic acid profiles was found in leaves of Wyalkatchem (Supplementary Table S3). Itaconate enhanced 1.6-fold in Wyalkatchem leaves under low P. In roots, the levels of malic acid increased (1.3-fold) in Wyalkatchem under low P. P deficiency led to increases in galactonic acid (1.3-fold), gluconate (1.4-fold) and glyceric acid (1.4-fold) in roots of Wyalkatchem, while low P did not affect the levels of these organic acids in roots of RAC875 (Supplementary Table S3). Low P resulted in the enhancement of shikimic acid, a precursor for aromatic compounds in leaves of both RAC875 (1.6-fold) and Wyalkatchem (1.2-fold), while no effect on this organic acid was found in roots under low P (Figure 1).

The abundance of a few amino acids reduced under low P, but the levels of most amino acids remained unchanged in shoots and roots under low P in both wheat genotypes (Figure 1 and Supplementary Table S3). For example, in leaves, N-acetyl serine and pyroglutamic acid significantly declined 0.6 and 0.5-fold in RAC875, respectively; alanine and putrescine decreased 0.7-fold in Wyalkatchem. In roots, P deficiency significantly (*P* < 0.05) reduced levels of O-acetyl serine (0.8-fold) and threonine (0.8-fold) in Wyalkatchem and serine (0.7-fold) in RAC875.

### Variability in Metabolite Profiles of Two Genotypes in Response to P Deficiency

**FIGURE 2:**
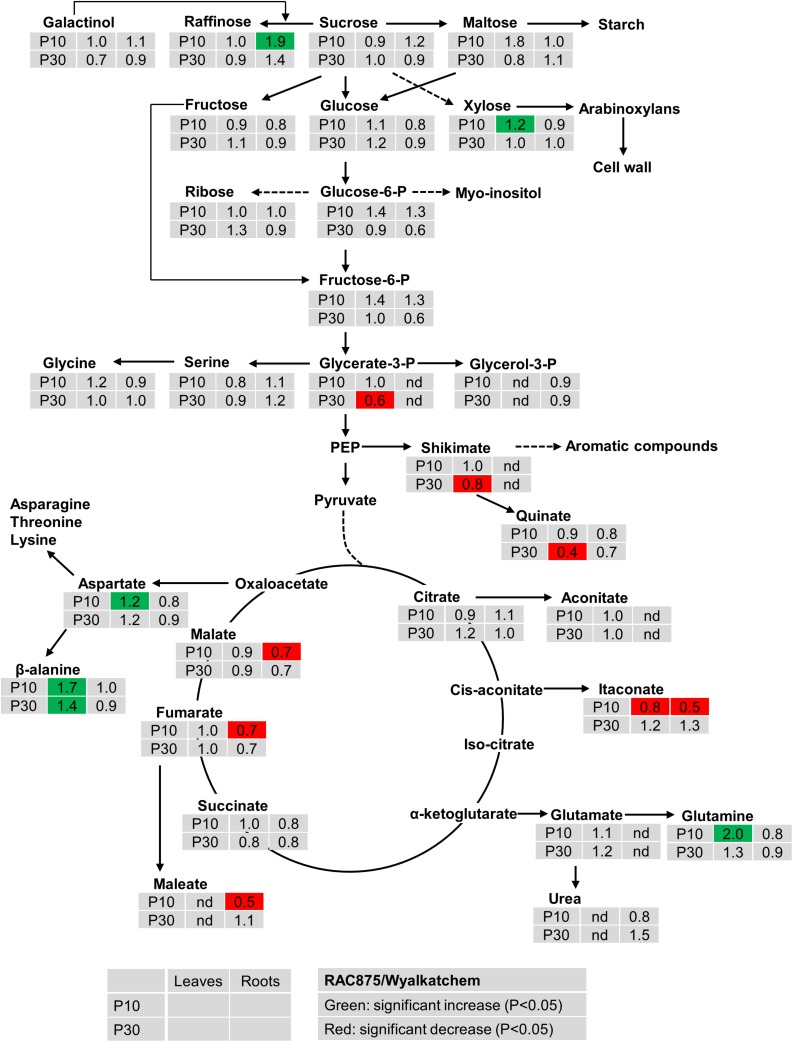
Variation in the levels of metabolites from shoots and roots between two wheat genotypes RAC875 and Wyalkatchem at 28 days after sowing (DAS). Plants were grown in sandy soils at low (10 mg P kg^–1^ soil – P10) and adequate (30 mg P kg^–1^ soil – P30) P supply. Relative ratios between RAC875 and Wyalkatchem in shoots (the first column) and roots (the second column) are presented as means of four biological replicates and relative ratios were compared at low P (P10) (the first row) and adequate P (P30) (the second row). Significant increases (*P* < 0.05) are indicated in green and significant decreases (*P* < 0.05) are indicated in red; nd, not detectable.

Under both levels of P supply, leaf sucrose, maltose, raffinose, glucose and fructose were not significantly (*P* < 0.05) different between the two wheat genotypes (Figure 2). Under low P supply, RAC875 had a greater leaf xylose level (1.2-fold) but lower leaf galactonic acid (an oxidized sugar) level (0.7-fold) when compared to Wyalkatchem (Supplementary Table S4). In roots, RAC875 had significantly higher levels of only 1-kestose and raffinose (both 1.9-fold) when compared to Wyalkatchem under low P (Figure 2).

There were no significant differences in two phosphorylated sugars (glucose-6-P, fructose-6-P) between two wheat genotypes in both leaves and roots under both low and adequate P (Figure 2). In leaves, no genotypic variation occurred in glycerol-3-P under P deficiency, but the level of glycerol-3-P in RAC875 was 0.6 times lower than in Wyalkatchem under adequate P (Figure 2). However, in roots, glycerol-3-P were not detected under both P treatments.

The levels of many organic acids were lower (*P* < 0.05) in roots of RAC875 (itaconate, 0.5-fold, maleate 0.5-fold, galactonic 0.6, fumaric 0.7-fold, and malate 0.7-fold) than in Wyalkatchem under P deficiency (Figure 2 and Supplementary Table S4). However, under low P the levels of these organic acids in leaves were not significantly (*P* < 0.05) different between the two wheat genotypes.

Genotypic variation was found in the levels of several amino acids. Under low P, there was an increase in aspartic acid, beta-alanine and glutamine of 1.2, 1.7, and 2.0 fold (respectively) in RAC875 when compared to Wyalkatchem (Figure 2). However, no significant differences (*P* < 0.05) were found in amino acids levels between the two wheat genotypes under adequate P.

### Organic Acid in Root Exudates

#### The Recovery of Organic Acids Extracted From Root Exudates

**FIGURE 3:**
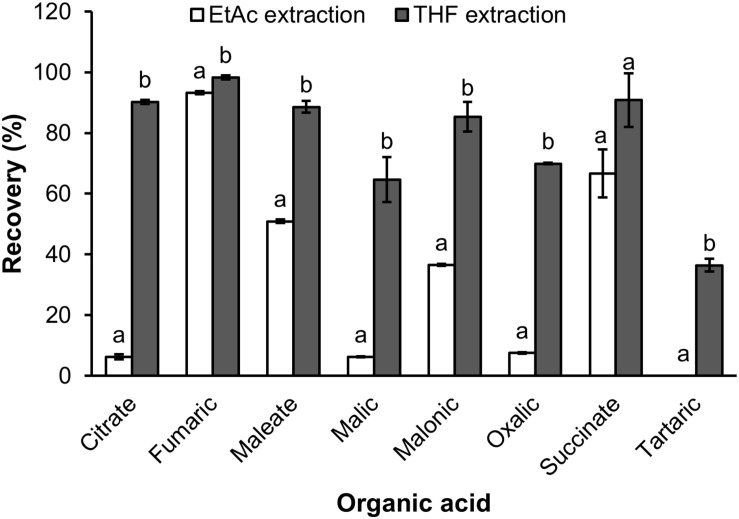
The recovery of organic acids extracted by ethyl acetate (EtAc) and tetrahydrofuran (THF) from milliQ water spiked organic acids. Data represents the mean and standard error of three biological replicates. Different letters show significant differences (*P* < 0.05).

**FIGURE 4:**
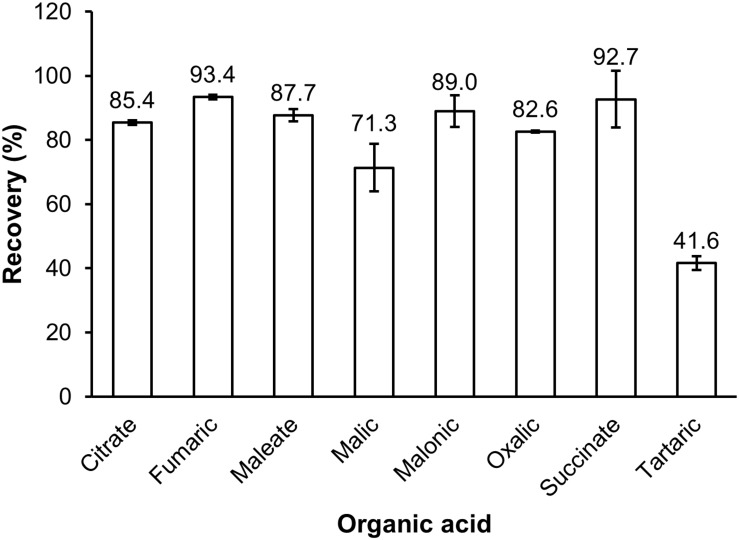
The recovery of organic acids extracted by THF from soil solution spiked organic acids. Data represents the mean and standard error of four biological replicates.

The recovery of organic extraction was presented in Figure 3. The results showed that THF had greater capacity of organic extraction than EtAc of the test solution. For THF the recovery ranged from 34.6% for tartaric acid to 98.3% for fumaric acid, whilst the recovery by EtAc ranged from 0 for tartaric acid to 90.2 for fumaric acid (Figure 3). THF had a 9.3, 10.4, and 14.5 fold higher recovery of oxalic, malic and citric acid than EtAc, respectively. THF also showed a 1.7 and 2.3 fold greater recovery of maleic and malonic acid than EtAc, respectively. The recovery of the eight organic acids was also confirmed in a sample of soil solution spiked with a mix of the eight standards and extracted with THF. The results showed that the recoveries of organic acids extracted from the soil solution spiked organic acids were similar to those extracted from the original mix of organic acids (Figures 3, 4).

#### Changes in Organic Acid Concentration in Root Exudates From Two Wheat Genotypes at 41 DAS Under Different P Levels

Four organic acids including succinic, maleic, oxalic and citric were identified in the root exudates of the wheat genotypes, while malic, malonic, tartaric, and fumaric acids were not detected. The results from Supplementary Table S3 showed that P supply significantly (*P* < 0.05) affected maleic and succinic acid concentrations, but no significant effects were observed on oxalic and citric acid concentrations (*P* = 0.242, *P* = 0.285 respectively).

**FIGURE 5:**
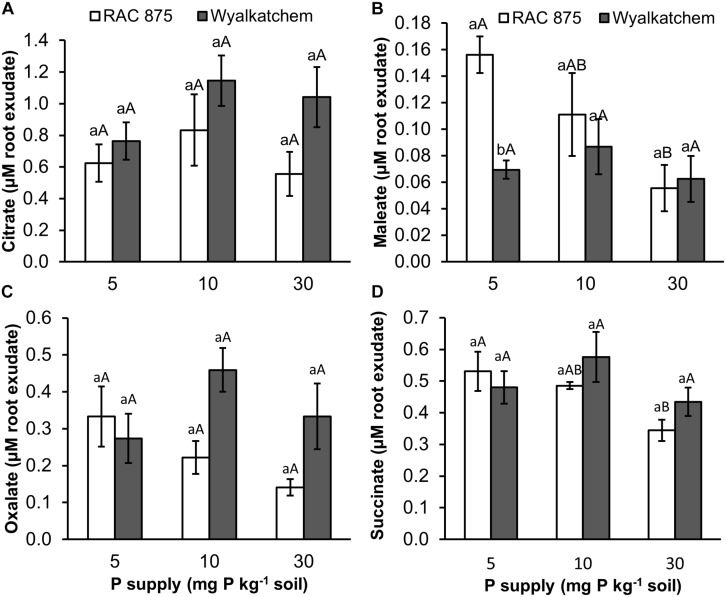
Organic acid (**A** = citrate, **B** = maleate, **C** = oxalate, and **D** = succinate) secretion in root exudate of two wheat genotypes RAC 875 and Wyalkatchem grown under different P treatments. Data represents the mean and standard error of five biological replicates. Different small letters **(a,b)** show significantly different between genotypes within the same P supply (*P* < 0.05); different capital letters **(A,B)** show significantly different between P supply in each genotype (*P* < 0.05).

The two wheat genotypes showed differences in organic acid secretion when responding to P deficiency. Under low P (10 mg P kg^–1^ soil), RAC875 released higher levels of maleic, oxalic, and succinic acids in root exudates when compared to adequate P (30 mg P kg^–1^ soil). Under very low P (5 mg P kg^–1^ soil), concentrations of maleic, oxalic and succinic acids were 2.8, 2.4, and 1.5 fold higher respectively, than these organic acid concentrations under adequate P with *P* values of 0.027, 0.064, and 0.026 respectively (Figure 5 and Supplementary Tables S5–S7). However, P supply did not affect these root exudate, organic acid concentrations in Wyalkatchem (Supplementary Table S4).

## Discussion

### P Deficiency Enhances Levels of Several Sugars and the P Efficient Wheat Genotype (RAC875) Accumulated Greater Raffinose

Although P deficiency resulted in increased levels of several sugars such as raffinose, galactinol, 1-kestose and xylose, sugar responses to low P were not uniform between leaves and roots, nor between the two wheat genotypes. Indeed, P deficiency strongly increased raffinose in roots of both wheat genotypes (more so in RAC875), while no changes in this sugar was observed in leaves (Figure 1 and Supplementary Figures S1, S2). Under low P, an increase in galactinol was found only in leaves of RAC875. Significant increases in xylose were only found in leaves of RAC875 and roots of Wyalkatchem. Low P led to the enhancement of 1-kestose in roots of both wheat genotypes, however, this metabolite was undetectable in leaves. Differences in sugar responses between shoots and roots to drought stress have also been observed in the common C3 grasses *A. pratensis* and *H. lanatus* (Gargallo-Garriga et al., 2014).

The higher level of 1-kestose is interesting as it is implicated in abiotic stress tolerance through accumulation as a reserve carbohydrate and also as being involved in membrane stability under abiotic stress [Valluru and Van den Ende (2008) and references therein]. Raffinose is also interesting as it belongs to the raffinose family oligosaccharides (RFOs) which function as stored carbohydrates and stress tolerance factors (Van den Ende, 2013). Our observation of an increase of raffinose in roots under low P is consistent with a study in tomato (Sung et al., 2015). Increased levels of raffinose under low P have also been documented in barley (Huang et al., 2008) and maize (Ganie et al., 2015). The accumulation of raffinose in plants has been found under stress conditions such as iron deficiency (Rellán-Álvarez et al., 2010), cold (Rohloff et al., 2012), drought (Peters et al., 2007), and salinity (Shelden et al., 2016). Interestingly, RFOs are derived from Suc and synthesized through the addition of active Gal moieties donated by galactinol (Peterbauer and Richter, 2001). Both galactinol and raffinose have been shown to protect plants from oxidative stress (Nishizawa et al., 2008). In *Arabidopsis thaliana*, it appears that both galactinol and raffinose not only act as osmoprotectants and stabilizers of cellular membranes, but also as scavengers of reactive oxygen species (ROS), and that they play a role in the protection of cellular metabolism, in particular, the photosynthesis of chloroplasts (Nishizawa et al., 2008). Therefore, the accumulation of raffinose and galactinol is most likely the adaptive mechanism of RAC875 to low P supply. It would be interesting to further look at this apparent efficiency trait in a mapping population, where RAC875 is a donor parent. It is possible that even more efficient lines could be derived through transgressive segregation and they in-turn could be used as donor parents in the breeding of P efficient wheat.

Contrary to previously reported studies that sucrose and maltose increased under low P as in barley (Huang et al., 2008) and in maize (Ganie et al., 2015) or other plants (Ciereszko and Barbachowska, 2000; Ciereszko et al., 2002), this study shows that P supply had no significant impact on the levels of these sugars (Figure 1). This difference could be due to different cultivating methods and the harvest stages (i.e., in this study, plants were grown in sandy soil and harvested at 28 DAS, while Huang et al. (2008) and Ganie et al. (2015) used hydroponic cultivation and harvested at earlier stages). The level of deficiency could also lead to different results. Indeed, in the experiment carried out by Huang et al. (2008), barley plants were grown under severe P deficiency. Genotypic variation may result in differences in metabolic responses to low P. For example, sucrose levels significantly dropped in the leaves of a *Brachiaria* hybrid under low P but not in rice (Nanamori et al., 2004). Growth stages also lead to differences in sugar responses to low P. For instance, Muller et al. (2015) were able to show that low P reduced the levels of sucrose, glucose and fructose in lupin shoot after 14 days of P deficiency, but no changes in these sugars were found after 22 days of P deficiency. Glucose and fructose levels were also found to stay unchanged under low P deprivation in this study.

### Maintaining Phosphorylated Sugars in the P Efficient Wheat

Adjustment of phosphorylated sugars is important for plants to adapt to P deficiency since these are intermediate metabolites of metabolic processes. In this study, glucose-6-P and fructose-6-P strongly declined in both leaves and roots of the low P intolerant wheat, Wyalkatchem under P deficiency (Figure 1). This result is consistent with previous studies. For example, a reduction in these metabolites was also observed in shoots and roots of barley (Huang et al., 2008) and of maize (Ganie et al., 2015). Low P also reduced the levels of phosphorylated sugars in bean roots (Rychter and Randall, 1994) and in leaves of *Eucalyptus globulus* (Warren, 2011). Glucose-6-P and fructose-6-P are important intermediates for glycolysis. Glycolysis is the first stage of carbohydrate metabolism, followed by the tricarboxylic acid cycle (TCA) in the cell. This process not only generates energy but also provides important intermediates for the biosynthesis of essential molecules (i.e., amino acids). Thus, the reduction in glucose-6-P and fructose-6-P would essentially lead to lower biomass production in Wyalkatchem under P deficiency.

In contrast to Wyalkatchem, low P had no effect on the levels of these phosphorylated sugars in the low P tolerant wheat, RAC875 (Figure 1). The maintenance of phosphorylated sugars in RAC875 under low P could maintain respiratory carbon flux to generate energy and carbon skeletons for key biochemical processes in plants, which supports RAC875 in producing a greater relative biomass under low P supply. Besides, the relative ratio of inorganic P (P_i_) in leaves between low P and adequate P was higher in RAC875 (0.8-fold) than in Wyalkatchem (0.5-fold) (Supplementary Table S3), indicating that under low P, RAC875 can maintain relatively high P_i_ levels. This enables RAC875 to maintain more efficient biochemical processes that requires P_i_. The high ratio of P_i_ in leaves (low P/adequate P) of RAC875 is correlated with the ratio of shoot P concentration at 24 DAS (low P/adequate P) which was 0.5-fold for RAC875 and 0.4-fold for Wyalkatchem (data unpublished).

When P supply is low, a drop in glycerol-3-P occurred in roots of both wheat genotypes (Figure 1). This result agrees with a report in lupin (Muller et al., 2015). Glycerol-3-P is a structural component of phospholipids that can be replaced with sulfo- and galactolipids under P deprivation (Plaxton and Tran, 2011; Lambers et al., 2012; Veneklaas et al., 2012). Therefore, it would be interesting to identify whether variation in sulfo-and galactolipids under P deficiency is present between the two wheat genotypes.

### Low Organic Acids of TCA Cycle but High Accumulation of Shikimic Acid and Quinic Acid in the P Efficient Wheat

The levels of most organic acids (i.e., citrate, succinate and fumarate) involved in the TCA cycle were not affected by P deficiency (Figure 1). This is similar to the results from *Eucalyptus globulus* (Warren, 2011). However, low P reduced the levels of succinate and fumarate in barley roots (Huang et al., 2008) and decreased succinate levels in maize leaves (Ganie et al., 2015), while low P enhanced the levels of citrate, succinate and fumarate in shoots and roots of lupin (Muller et al., 2015). Huang et al. (2008) suggests that decreased organic acid levels in barley roots under low P are related to the shortage of carbohydrate and the secretion of organic acids in response to the P starvation and this could reduce their levels. The drop in organic acids seems to be the main reason that hindered the TCA cycle in barley since plants was grown under severe P deficiency in the experiment carried out by Huang et al. (2008). Meanwhile, lupin forms cluster roots that produce high amounts of organic acids (Muller et al., 2015) and they can be secreted into the rhizosphere to respond to P starvation (Hocking and Jeffery, 2004; Cheng et al., 2014). In this study, no changes in citrate, succinate and fumarate under low P was observed, indicating that plants appear to maintain normal levels of carbohydrates for respiration.

However, low P enhanced aconitate and itaconate in leaves of Wyalkatchem and increased maleate in roots of Wyalkatchem. Low P strongly reduced the level of itaconate in roots of RAC875 under low P supply. Interestingly, under low P, RAC875, showed lower levels of fumarate, malate, maleate, and itaconate in roots when compared to Wyalkatchem (Figure 2). This may indicate that RAC875 requires lower levels of carbohydrates to maintain its normal carbohydrate metabolism, while Wyalkatchem needs higher levels of carbohydrates for its normal cellular activities. Higher secretion of organic acids to adapt to P deficiency may reduce in the levels of these organic acids in roots of RAC875.

Contrary to the stability of organic acids in the TCA cycle between P treatments, shikimic acid and quinic acid increased in leaves of RAC875 in response to P deficiency (Figure 1). Low P did not affect quinic acid in both shoots and roots of Wyalkatchem, but shikimic acid increased in leaves of Wyalkatchem. Under low P, increased level of shikimic acid were also observed in barley shoots (Huang et al., 2008) and in lupin shoots (Muller et al., 2015). Shikimic acid is important in the biosynthesis of aromantic compounds (Herrmann, 1995), while quinic acid is a side product of the shikimic pathway that may be used as a stored source for shikimic acid production (Marsh et al., 2009). Plants appear to produce secondary metabolites from shikimic acid to protect them from abiotic stress environments (Herms and Mattson, 1992; Rakhmankulova et al., 2003).

### The P Efficient Wheat Accumulated Several Amino Acids to a Higher Level

In contrast to increased levels of amino acids in barley and lupin under low P (Huang et al., 2008; Muller et al., 2015), in this study, low P had no effect on most of the amino acids and amines in both wheat genotypes (Figure 1, Supplementary Figures S1, S2, and Supplementary Tables S3, S4). Low P led to a significant decrease in N-acetyl serine and pyroglutamic acid for RAC875, and alanine and putrescine for Wyalkatchem. Our findings seem to agree with Arnold et al. (2015) that a lack of ATP could result in a decrease of amino acids and increase of sugars because P deficiency reduces ATP content in plant leaves (Mikulska et al., 1998; Carstensen et al., 2018). However, RAC875 showed significantly higher levels of aspartate, glutamine and β-alanine in leaves when compared to Wyalkatchem under low P supply. Aspartate is an important amino acid since it is a precursor for the biosynthesis of other amino acids (i.e., asparagine, lysine, methionine, isoleucine, and threonine) and other essential cellular compounds (pyrimidine and NAD) (Reitzer, 2004; Jander and Joshi, 2009), while glutamine is considered as a hub for nitrogen metabolism and functions as an amino group donor for cellular processes (Sheppard, 2015). Thus, increased accumulation of aspartate and glutamine would enable RAC875 to maintain metabolic activities under P deficiency. β-alanine is known for the biosynthesis of β-alanine betaine (Duhazé et al., 2003) which has a protective role for plants being exposed to abiotic stress (Singh et al., 2015). Higher β-alanine in RAC875 contributes to greater β-alanine betaine production which may lead to RAC875 being more tolerant to P deficiency.

### Low P Increased Levels of Several Organic Acids in the Root Exudates of the P Efficient Wheat RAC875

The presence of organic acids in soil solution enhances the solubility of P, particularly in complex forms and assists plant roots to absorb more P (Zhang et al., 1997). In response to P starvation, plants are known to release organic acids (Neumann and Römheld, 1999; Dotaniya et al., 2013). This study shows that the P efficient wheat, RAC875 increased secretion of malelic, oxalic, and succinic acids in response to changing P levels, while changes did not occur in Wylakatchem. Interestingly, under low P, RAC875 showed lower levels of fumarate, malate, maleate and itaconate in root tissues, when compared to Wyalkatchem (Figure 2). This agrees with a study in maize (Gaume et al., 2001), in which the low P intolerant genotype was characterized by high organic acid levels in roots and low organic acid secretion.

A limitation of this study is the absence of more phenotypic data that may also shed light on the root mechanisms involved in the efficiency mechanism. In a recent publication, Ziegler et al. (2016) were able to show a role of coumarin secretion and lignification in response to P deficiency. The authors detected a partial overlap between Pi and Fe-deficiency-induced changes in root exudate composition. RSA modifications could occur through changes in lignin deposition, with an inverse relationship between oligolignol content in root exudates, and lignin deposition witnessed. As *Arabidopsis* is a Strategy I plant and wheat is a Strategy II plant, Fe acquisition does vary and it would be interesting if similar mechanisms are at play between dicots and monocots for changes in RSA in response to low P.

## Conclusion

In general, this work has contributed toward our understanding of the P efficiency mechanism in the wheat genotype, RAC875. A major outcome is the role of metabolites known to reduce the adverse effects of ROS and also act as osmoprotectants. The efficient wheat, RAC875 accumulates higher levels of raffinose in roots and also maintains high levels of phosphorylated sugars (glucose-6-P and fructose-6-P) under low P, while P deficiency reduced these phosphorylated metabolites in both leaves, and roots of Wyalkatchem. 1-kestose also increased in RAC875 and this is implicated in stress tolerance. Organic acids of the TCA cycle (citrate, succinate, and fumarate) had no change under P deficiency, whereas at low P, shikimic acid and quinic acid increased in leaves of RAC875. RAC875 showed lower levels of fumarate, malate, maleate and itaconate in roots, when compared to Wyalkatchem under low P supply. In contrast, low P enhanced organic acid exudation in RAC875 and this may implicate these metabolites in the acidification of the rhizosphere and aid in availability of P from the soil solution. Several amino acids including aspartate, glutamine and β-alanine were accumulated greater in RAC875 leaves than in Wyalkatchem under low P. Taken together, a greater accumulation of raffinose and 1-kestose in roots, and an accumulation of aspartate, glutamine and β-alanine in leaves may contribute to a more P efficient RAC875. Maintaining levels of glucose-6-P and fructose-6-P would appear to maintain normal carbohydrate flux that is beneficial for the growth of RAC875 under P deficiency.

## Author Contributions

VN and JS designed the research. VN implemented the experiments, performed the data analyses, and wrote the manuscript. LP involved in the organic acid analysis. UR analyzed the metabolites. LP, UR, and JS made the revision of the manuscript. All authors approved the final version of the manuscript to be published.

## Conflict of Interest Statement

The authors declare that the research was conducted in the absence of any commercial or financial relationships that could be construed as a potential conflict of interest.

## Footnotes

1 http://www.nist.gov

2 http://gmd.mpimp-golm.mpg.de/

## Supplementary Material

FIGURE S1 Comparative metabolite analysis between shoot of Wyalkatchem and RAC875 grown at low P: 10 mg P kg^–1^ soil and adequate P: 30 mg P kg^–1^ soil. W30P, Wyalkatchem grown at 30 mg P kg^–1^ soil; W10P, Wyalkatchem grown at 10 mg P kg^–1^ soil; R30P, RAC875 grown at 30 mg P kg^–1^ soil; R10P, RAC875 grown at 10 mg P kg^–1^ soil.

FIGURE S2 Comparative metabolite analysis between root of Wyalkatchem and RAC875 grown at low P: 10 mg P kg^–1^ soil and adequate P: 30 mg P kg^–1^ soil. W30P, Wyalkatchem grown at 30 mg P kg^–1^ soil; W10P, Wyalkatchem grown at 10 mg P kg^–1^ soil; R30P, RAC875 grown at 30 mg P kg^–1^ soil; R10P, RAC875 grown at 10 mg P kg^–1^ soil.

TABLE S1 Basal nutrient mix (mg kg^–1^ soil) added to the growth medium.

TABLE S2 Separation conditions used for HPIC analysis of organic acids.

TABLE S3 Fold-change in metabolite levels in shoots of P efficient RAC875 and P inefficient Wyalkatchem. Comparisons between low P supply and adequate P supply (low P/adequate P); and between RAC875 and Wyalkatchem (RAC875/Wyalkatchem) at each P supply. R, RAC875; W, Wyalkatchem; Low P, 10 mg P kg^–1^ soil (P10), adequate P: 30 mg P kg^–1^ soil (P30); sem, standard error of the mean of 4 replicates. Blue color: significant at *P* < 0.05 (*t*-test).

TABLE S4 Fold-change in metabolite levels in roots of the P efficient RAC875 and P inefficient Wyalkatchem. Comparisons between low P supply and adequate P supply (low P/adequate P); and between RAC875 and Wyalkatchem (RAC875/Wyalkatchem) at each P supply. R, RAC875; W, Wyalkatchem; Low P, 10 mg P kg**^–^**^1^ soil (P10), adequate P: 30 mg P kg**^–^**^1^ soil (P30); sem, standard error of the mean of 4 replicates. Blue color: significant at *P* < 0.05 (*t*-test); green color: significant at *P* < 0.05 with Bonferroni correction.

TABLE S5 Effect of P supply on succinic, maleic, oxalic and citric acids of two wheat genotypes RAC875 and Wyalkatchem at 41 days after sowing (DAS).

TABLE S6 Effect of P supply on succinic, maleic, oxalic and citric acid concentrations of each wheat genotype, RAC875, and Wyalkatchem at 41 DAS (One-way Anova analysis).

TABLE S7 Comparisons for succinic, maleic, oxalic, and citric acids between P supply of each wheat genotype (one-way Anova, Turkey test).
