# Supplementary material for: Genotypic Variation in the Root and Shoot Metabolite Profiles of Wheat (Triticum aestivum L.) Indicate Sustained, Preferential Carbon Allocation as a Potential Mechanism in Phosphorus Efficiency
Source: Front Plant Sci. 2019 Aug 6;10:995. doi: 10.3389/fpls.2019.00995 (PMC6691131; doi:10.3389/fpls.2019.00995)
Supplement: TABLE S1 — Basal nutrient mix (mg kg–1 soil) added to the growth medium. [file Table_1.docx]

Supplementary Material

Genotypic variation in the root and shoot metabolite profiles of wheat (*Triticum aestivum*) indicate sustained, preferential carbon allocation as a potential mechanism in phosphorus efficiency

Van Lam Nguyen^1, 2^, Lachlan Palmer^1^, Ute Roessner^3^ and James Stangoulis^1*^

^1^College of Science and Engineering, Flinders University, Sturt Rd, Bedford Park SA 5042, Australia.

^2^Department of Biochemistry and Food Biotechnology, Vietnam National University of Agriculture, Trau Quy, Gia Lam, Hanoi, Vietnam.

^3^School of BioSciences, The University of Melbourne, 3010 Victoria, Australia.

**^*^Correspondence:** James Stangoulis

Email: [james.stangoulis@flinders.edu.au](mailto:james.stangoulis@flinders.edu.au)

Postal address: GPO Box 2100, Adelaide 5001, South Australia

Table S 1. Basal nutrient mix (mg kg^-1^ soil) added to the growth medium

| Nutrient | Concentration |
| --- | --- |
| Ca(NO_3_)_2_.4H_2_O | 918 |
| K_2_SO_4_ | 113.6 |
| MgSO_4_.7H_2_O | 140 |
| FeSO_4_.7H_2_O | 1.4 |
| Na_2_MoO_4_.2H_2_O | 0.61 |
| CuSO_4_.5H_2_O | 2.25 |
| MnSO_4_.4H_2_O | 3.68 |
| ZnSO_4_.7H_2_O | 6.6 |
| H_3_BO_3_ | 0.28 |
| KH_2_PO_4_ | 5*, 10, 30 |

*only used for the exudate experiment.

Table S 2. Separation conditions used for HPIC analysis of organic acids

| Time (min) | KOH (mM) | Methanol (%) | Comment |
| --- | --- | --- | --- |
| 0 | 1 | 10 | Injection, analysis start |
| 8 | 1 | 10 | End of isocratic section, start of first gradient |
| 18 | 15 | 20 | End of first gradient, start of second gradient |
| 28 | 30 | 20 | End of second gradient, start of third gradient |
| 38 | 60 | 10 | End of third gradient |
| 38 | 100 | 10 | Start of column wash |
| 42 | 100 | 10 | End of column wash |
| 42 | 1 | 10 | Start of equilibration |
| 47 | 1 | 10 | End of equilibration, end of run |

Table S 3. Fold-change in metabolite levels in leaves of efficient (RAC875) and inefficient (Wyalkatchem). Comparisons between low P supply and adequate P supply (low P/adequate P); and between RAC875 and Wyalkatchem (RAC875/Wyalkatchem) at each P supply. R: RAC875; W: Wyalkatchem; Low P: 10 mg P kg^-1^ soil (P10), adequate P: 30 mg P kg^-1^ soil (P30); sem: standard error of the mean (n=4 biological replications). * significant at P<0.05 (t-test).

| Metabolite | Low P/adequate P | | | | RAC875/Wyalkatchem | | | |
| --- | --- | --- | --- | --- | --- | --- | --- | --- |
|  | RAC875 | | Wyalkatchem | | P10 | | P30 | |
|  | P10/ P30 | sem | P10/ P30 | sem | R/W | sem | R/W | sem |
| Amino Acids and Amines |  |  |  |  |  |  |  |  |
| 4-amino-butyric acid | 0.431 | 0.332 | 0.653 | 0.245 | 1.274 | 0.332 | 1.930 | 0.236 |
| Adenine | 0.791 | 0.071 | 0.856 | 0.118 | 1.097 | 0.071 | 1.188 | 0.074 |
| Alanine | 0.931 | 0.089 | 0.700* | 0.112 | 1.174 | 0.089 | 0.883 | 0.047 |
| Aspartic acid | 0.805 | 0.024 | 0.843 | 0.058 | 1.180* | 0.024 | 1.236 | 0.134 |
| beta-alanine | 1.061 | 0.113 | 0.867 | 0.159 | 1.727* | 0.113 | 1.410* | 0.012 |
| Cytosine | 0.922 | 0.102 | 1.270 | 0.074 | 0.883 | 0.102 | 1.217 | 0.096 |
| Ethanolamine | 0.987 | 0.218 | 0.795 | 0.077 | 1.010 | 0.218 | 0.813 | 0.058 |
| Glutamic acid | 0.878 | 0.085 | 0.981 | 0.083 | 1.066 | 0.085 | 1.190 | 0.150 |
| Glutamine | 0.724 | 0.185 | 0.489 | 0.170 | 1.958* | 0.185 | 1.322 | 0.145 |
| Glycine | 0.790 | 0.150 | 0.644 | 0.154 | 1.185 | 0.150 | 0.966 | 0.223 |
| Guanine | 1.211 | 0.082 | 1.133 | 0.036 | 1.027 | 0.082 | 0.961 | 0.078 |
| Guanosine | 1.102 | 0.057 | 0.768 | 0.067 | 1.418* | 0.057 | 0.988 | 0.061 |
| Homoserine | 0.945 | 0.218 | 0.702 | 0.212 | 1.145 | 0.218 | 0.850 | 0.303 |
| Isoleucine | 1.145 | 0.238 | 0.904 | 0.252 | 1.066 | 0.238 | 0.842 | 0.215 |
| N-acetyl serine | 0.589* | 0.064 | 0.912 | 0.143 | 0.695 | 0.064 | 1.075 | 0.124 |
| Phenylalanine | 1.026 | 0.066 | 0.977 | 0.116 | 0.848 | 0.066 | 0.807 | 0.110 |
| Proline | 0.812 | 0.527 | 0.795 | 0.354 | 1.772 | 0.527 | 1.735 | 0.327 |
| Putrescine | 0.856 | 0.137 | 0.698* | 0.075 | 0.780 | 0.137 | 0.636* | 0.089 |
| Pyroglutamic acid | 0.529* | 0.093 | 0.560 | 0.293 | 0.848 | 0.093 | 0.897 | 0.058 |
| Serine | 0.704 | 0.236 | 0.792 | 0.191 | 0.828 | 0.236 | 0.932 | 0.260 |
| Threonine | 0.775 | 0.083 | 0.715 | 0.118 | 1.172 | 0.083 | 1.081 | 0.096 |
| Uracil | 0.719 | 0.114 | 1.036 | 0.131 | 0.854 | 0.114 | 1.231 | 0.065 |
| Valine | 0.925 | 0.150 | 0.829 | 0.148 | 1.114 | 0.150 | 0.999 | 0.179 |
| Organic Acids |  |  |  |  |  |  |  |  |
| 2- oxo-glutaric acid | 1.259 | 0.086 | 0.778 | 0.107 | 1.288 | 0.086 | 0.796 | 0.090 |
| 2-methyl Maleic acid/Fumaric acid | 0.854 | 0.043 | 1.240 | 0.049 | 0.909 | 0.043 | 1.319 | 0.079 |
| 4-hydroxy-benzoic acid | 0.607* | 0.095 | 1.059 | 0.140 | 0.758 | 0.095 | 1.322 | 0.035 |

Table S 3. Fold-change in metabolite levels in leaves of efficient (RAC875) and inefficient (Wyalkatchem). Comparisons between low P supply and adequate P supply (low P/adequate P); and between RAC875 and Wyalkatchem (RAC875/Wyalkatchem) at each P supply. R: RAC875; W: Wyalkatchem; Low P: 10 mg P kg^-1^ soil (P10), adequate P: 30 mg P kg^-1^ soil (P30); sem: standard error of the mean (n=4 biological replications). Blue color: significant at P<0.05 (t-test) (continue)

| Metabolite | Low P/adequate P | | | | RAC875/Wyalkatchem | | | |
| --- | --- | --- | --- | --- | --- | --- | --- | --- |
|  | RAC875 | | Wyalkatchem | | P10 | | P30 | |
|  | P10/ P30 | sem | P10/ P30 | sem | R/W | sem | R/W | sem |
| Aconitic acid | 1.334* | 0.039 | 1.304* | 0.053 | 0.985 | 0.039 | 0.963 | 0.088 |
| Benzoic acid | 0.878* | 0.029 | 0.990 | 0.046 | 0.975 | 0.029 | 1.099 | 0.028 |
| Caffeic acid | 0.941 | 0.067 | 1.147 | 0.152 | 0.823 | 0.067 | 1.003 | 0.017 |
| Citric acid | 0.714 | 0.133 | 0.920 | 0.155 | 0.897 | 0.133 | 1.156 | 0.262 |
| Ferulic acid | 1.094 | 0.143 | 1.232 | 0.151 | 1.129 | 0.143 | 1.271 | 0.070 |
| Aconitic acid | 1.334* | 0.039 | 1.304* | 0.053 | 0.985 | 0.039 | 0.963 | 0.088 |
| Benzoic acid | 0.878* | 0.029 | 0.990 | 0.046 | 0.975 | 0.029 | 1.099 | 0.028 |
| Caffeic acid | 0.941 | 0.067 | 1.147 | 0.152 | 0.823 | 0.067 | 1.003 | 0.017 |
| Citric acid | 0.714 | 0.133 | 0.920 | 0.155 | 0.897 | 0.133 | 1.156 | 0.262 |
| Ferulic acid | 1.094 | 0.143 | 1.232 | 0.151 | 1.129 | 0.143 | 1.271 | 0.070 |
| Fumaric acid | 1.205 | 0.074 | 1.231 | 0.056 | 0.976 | 0.074 | 0.997 | 0.053 |
| Glyceric acid | 0.997 | 0.062 | 1.100 | 0.079 | 1.025 | 0.062 | 1.131 | 0.111 |
| Glyceric acid-3-phosphate | 1.172 | 0.188 | 0.685 | 0.252 | 0.991 | 0.188 | 0.579* | 0.190 |
| Glycolic acid | 0.909 | 0.032 | 1.104 | 0.033 | 0.941 | 0.032 | 1.143 | 0.045 |
| Iso Citric | 1.228* | 0.051 | 1.055 | 0.069 | 1.125 | 0.051 | 0.966 | 0.051 |
| Itaconate | 0.953 | 0.082 | 1.579* | 0.065 | 0.750* | 0.082 | 1.243 | 0.115 |
| Malic acid | 1.150 | 0.046 | 1.144 | 0.039 | 0.940 | 0.046 | 0.936 | 0.110 |
| Malonic acid | 1.647* | 0.172 | 1.185 | 0.123 | 1.032 | 0.172 | 0.743 | 0.075 |
| Oxalic acid | 1.203 | 0.275 | 1.247 | 0.239 | 1.041 | 0.275 | 1.079 | 0.236 |
| Qunic acid | 2.861* | 0.191 | 1.226 | 0.230 | 0.899 | 0.191 | 0.385* | 0.191 |
| Saccharic acid | 1.621* | 0.135 | 1.166 | 0.174 | 1.064 | 0.135 | 0.765 | 0.107 |
| Shikimic acid | 1.555* | 0.019 | 1.178* | 0.041 | 0.999 | 0.019 | 0.757* | 0.060 |
| Sinapic acid | 0.886 | 0.060 | 1.119 | 0.082 | 0.647* | 0.060 | 0.818 | 0.145 |
| Succinic acid | 1.023 | 0.071 | 0.848 | 0.108 | 0.965 | 0.071 | 0.800 | 0.075 |
| Threonic acid | 1.199 | 0.093 | 0.939 | 0.160 | 1.101 | 0.093 | 0.862 | 0.055 |
| Sugars, Sugar Alcohol and Sugar Phosphates |  |  |  |  |  |  |  |  |
| Arabinose | 1.015 | 0.043 | 1.101 | 0.029 | 0.945 | 0.043 | 1.025 | 0.009 |
| Arabitol | 0.746 | 0.187 | 1.197 | 0.143 | 0.969 | 0.187 | 1.554* | 0.124 |
| Beta Gentibiose | 1.032 | 0.178 | 1.099 | 0.088 | 1.111 | 0.178 | 1.183 | 0.097 |
| Erythritol | 0.894 | 0.078 | 1.126 | 0.122 | 1.127 | 0.078 | 1.419* | 0.037 |
| Fructose | 0.893 | 0.306 | 1.047 | 0.340 | 0.935 | 0.306 | 1.096 | 0.330 |

Table S 3. Fold-change in metabolite levels in leaves of efficient (RAC875) and inefficient (Wyalkatchem). Comparisons between low P supply and adequate P supply (low P/adequate P); and between RAC875 and Wyalkatchem (RAC875/Wyalkatchem) at each P supply. R: RAC875; W: Wyalkatchem; Low P: 10 mg P kg^-1^ soil (P10), adequate P: 30 mg P kg^-1^ soil (P30); sem: standard error of the mean (n=4 biological replications). * significant at P<0.05 (t-test) (continue)

| Metabolite | Low P/adequate P | | | | RAC875/Wyalkatchem | | | |
| --- | --- | --- | --- | --- | --- | --- | --- | --- |
|  | RAC875 | | Wyalkatchem | | P10 | | P30 | |
|  | P10/ P30 | sem | P10/ P30 | sem | R/W | sem | R/W | sem |
| Fructose-6-phosphate | 0.774 | 0.275 | 0.581* | 0.201 | 1.381 | 0.275 | 1.036 | 0.225 |
| Galactinol | 1.306* | 0.039 | 0.884 | 0.089 | 1.107 | 0.039 | 0.749 | 0.110 |
| Galactitol | 0.781 | 0.234 | 0.967 | 0.157 | 0.725 | 0.234 | 0.898 | 0.199 |
| Galactonic acid | 0.878 | 0.123 | 0.992 | 0.019 | 0.720* | 0.123 | 0.813* | 0.071 |
| Galactose | 0.990 | 0.191 | 1.177 | 0.144 | 0.788 | 0.191 | 0.936 | 0.100 |
| Gluconate | 0.956 | 0.149 | 1.041 | 0.149 | 1.114 | 0.149 | 1.212 | 0.094 |
| Gluconate-6-phosphate | 1.603 | 0.388 | 0.620 | 0.331 | 1.254 | 0.388 | 0.485 | 0.468 |
| Glucose | 0.980 | 0.191 | 1.012 | 0.256 | 1.134 | 0.191 | 1.171 | 0.224 |
| Glucose-6-phosphate | 0.875 | 0.295 | 0.560 | 0.243 | 1.353 | 0.295 | 0.865 | 0.168 |
| Maltose | 1.433 | 0.269 | 0.623 | 0.204 | 1.840 | 0.269 | 0.800 | 0.384 |
| Manitol | 0.643 | 0.106 | 0.858 | 0.319 | 0.397 | 0.106 | 0.530 | 0.177 |
| Manose | 0.894 | 0.144 | 0.861 | 0.159 | 1.185 | 0.144 | 1.141 | 0.076 |
| myo-Inositol | 0.983 | 0.038 | 1.018 | 0.101 | 1.062 | 0.038 | 1.099 | 0.062 |
| Raffinose | 1.101 | 0.097 | 0.972 | 0.310 | 1.031 | 0.097 | 0.910 | 0.176 |
| Ribitol | 0.981 | 0.035 | 1.006 | 0.060 | 0.941 | 0.035 | 0.965 | 0.070 |
| Ribose | 0.765* | 0.065 | 0.975 | 0.131 | 1.029 | 0.065 | 1.311 | 0.088 |
| Sucrose | 0.960 | 0.070 | 0.979 | 0.155 | 0.945 | 0.070 | 0.964 | 0.114 |
| Threitol | 0.755 | 0.191 | 1.086 | 0.152 | 1.232 | 0.191 | 1.773* | 0.134 |
| Trehelose | 1.254 | 0.034 | 0.884 | 0.093 | 1.248 | 0.034 | 0.880 | 0.098 |
| Xylitol | 0.756 | 0.148 | 0.961 | 0.092 | 1.374 | 0.148 | 1.746* | 0.090 |
| Xylose | 1.336* | 0.061 | 1.138 | 0.031 | 1.248* | 0.061 | 1.063 | 0.019 |
| Others |  |  |  |  |  |  |  |  |
| Glycerol | 1.139 | 0.096 | 0.595 | 0.102 | 0.887 | 0.096 | 0.464* | 0.144 |
| Hexadecanoic acid | 1.185 | 0.070 | 1.047 | 0.054 | 1.082 | 0.070 | 0.956 | 0.022 |
| Monomethylphosphate | 0.851 | 0.098 | 0.867 | 0.165 | 0.996 | 0.098 | 1.015 | 0.056 |
| Octadecanoic acid | 1.056 | 0.033 | 1.043 | 0.027 | 1.050 | 0.033 | 1.036 | 0.035 |
| Octadecatrienoic acid | 2.227* | 0.160 | 1.312 | 0.201 | 1.024 | 0.160 | 0.603 | 0.142 |
| Phosphoric acid | 0.844 | 0.184 | 0.526 | 0.297 | 1.189 | 0.184 | 0.740 | 0.236 |
| Threonic acid-1,4-lactone | 0.918 | 0.052 | 0.856 | 0.051 | 0.996 | 0.052 | 0.929 | 0.118 |

Table S 4. Fold-change in metabolite levels in roots of efficient (RAC875) and inefficient (Wyalkatchem). Comparisons between low P supply and adequate P supply (low P/adequate P); and between RAC875 and Wyalkatchem (RAC875/Wyalkatchem) at each P supply. R: RAC875; W: Wyalkatchem; Low P: 10 mg P kg-1 soil (P10), adequate P: 30 mg P kg-1 soil (P30); sem: standard error of the mean (n=4 biological replications). * significant at P<0.05 (t-test); ** significant at P<0.05 with Bonferroni correction.

| Metabolite | | Low P/adequate P | | | | RAC875/Wyalkatchem | | | |
| --- | --- | --- | --- | --- | --- | --- | --- | --- | --- |
|  |  | RAC875 | | Wyalkatchem | | P10 | | P30 | |
|  |  | P10/ P30 | sem | P10/ P30 | sem | R/W | sem | R/W | sem |
| Amino Acids and Amines |  | |  |  |  |  |  |  |  |
| Alanine | 0.881 | | 0.144 | 1.009 | 0.097 | 0.720 | 0.144 | 0.825 | 0.132 |
| Alanine, beta- | 1.269 | | 0.051 | 1.122 | 0.040 | 0.996 | 0.051 | 0.881 | 0.133 |
| Allantoin | 1.204 | | 0.182 | 2.108* | 0.128 | 0.874 | 0.182 | 1.531 | 0.219 |
| Asparagine | 0.772 | | 0.274 | 1.057 | 0.236 | 0.901 | 0.274 | 1.233 | 0.190 |
| Aspartic acid | 0.795 | | 0.108 | 0.889 | 0.061 | 0.804 | 0.108 | 0.899 | 0.140 |
| Butyric acid, 4-amino- | 0.891 | | 0.092 | 0.958 | 0.067 | 1.223 | 0.092 | 1.314 | 0.171 |
| Cytosine | 0.871 | | 0.031 | 0.945 | 0.064 | 0.915 | 0.031 | 0.993 | 0.124 |
| Ethanolamine | 0.822 | | 0.152 | 0.853 | 0.074 | 1.088 | 0.152 | 1.129 | 0.199 |
| Glutamine | 0.818 | | 0.552 | 0.913 | 0.188 | 0.829 | 0.552 | 0.925 | 0.357 |
| Glycine | 0.979 | | 0.050 | 1.128 | 0.057 | 0.898 | 0.050 | 1.034 | 0.117 |
| Guanine | 0.867 | | 0.118 | 1.180 | 0.094 | 0.912 | 0.118 | 1.241 | 0.145 |
| Guanosine | 1.756 | | 0.173 | 1.261 | 0.173 | 1.098 | 0.173 | 0.788 | 0.218 |
| Homoserine | 0.976 | | 0.105 | 0.946 | 0.070 | 1.052 | 0.105 | 1.021 | 0.162 |
| Isoleucine | 1.013 | | 0.068 | 1.084 | 0.074 | 0.914 | 0.068 | 0.978 | 0.187 |
| Leucine | 1.008 | | 0.105 | 1.171 | 0.054 | 0.914 | 0.105 | 1.062 | 0.188 |
| Lysine | 1.009 | | 0.109 | 1.183 | 0.109 | 0.930 | 0.109 | 1.091 | 0.180 |
| O-acetyl Serine | 0.712 | | 0.116 | 0.794* | 0.073 | 1.102 | 0.116 | 1.228 | 0.111 |
| Phenylalanine | 1.067 | | 0.047 | 1.057 | 0.048 | 1.014 | 0.047 | 1.005 | 0.155 |
| Proline | 0.911 | | 0.153 | 0.844 | 0.072 | 1.078 | 0.153 | 0.999 | 0.137 |
| Putrescine | 0.936 | | 0.165 | 0.762 | 0.206 | 0.869 | 0.165 | 0.708 | 0.164 |
| Pyroglutamic acid | 0.892 | | 0.114 | 0.747 | 0.060 | 1.348 | 0.114 | 1.130 | 0.152 |
| Serine | 0.745* | | 0.090 | 0.815 | 0.055 | 1.117 | 0.090 | 1.221 | 0.090 |
| Threonine | 0.801 | | 0.061 | 0.835* | 0.046 | 1.064 | 0.061 | 1.110 | 0.115 |
| Tyrosine | 0.926 | | 0.105 | 0.924 | 0.080 | 1.085 | 0.105 | 1.083 | 0.184 |
| Uracil | 0.478* | | 0.167 | 1.055 | 0.222 | 0.565 | 0.167 | 1.247 | 0.167 |
| Valine | 0.914 | | 0.090 | 0.938 | 0.056 | 1.071 | 0.090 | 1.099 | 0.136 |
| Organic Acids |  | |  |  |  |  |  |  |  |
| 4-Hydroxycinnamic acid | 0.800 | | 0.088 | 1.091 | 0.074 | 0.888 | 0.088 | 1.211 | 0.145 |
| Citric acid | 1.199 | | 0.046 | 1.082 | 0.064 | 1.076 | 0.046 | 0.972 | 0.067 |
| Ferulic acid, trans- | 0.652 | | 0.097 | 1.122 | 0.093 | 0.667* | 0.097 | 1.148 | 0.139 |
| Fumaric acid | 1.075 | | 0.110 | 1.079 | 0.057 | 0.704* | 0.110 | 0.706 | 0.164 |
| Galactonic acid | 1.070 | | 0.053 | 1.342* | 0.015 | 0.600 ^**^ | 0.053 | 0.752 | 0.090 |
| Gluconate | 1.042 | | 0.101 | 1.368* | 0.056 | 0.848 | 0.101 | 1.113 | 0.206 |

Table S 4. Fold-change in metabolite levels in roots of efficient (RAC875) and inefficient (Wyalkatchem). Comparisons between low P supply and adequate P supply (low P/adequate P); and between RAC875 and Wyalkatchem (RAC875/Wyalkatchem) at each P supply. R: RAC875; W: Wyalkatchem; Low P: 10 mg P kg^-1^ soil (P10), adequate P: 30 mg P kg^-1^ soil (P30); sem: standard error of the mean (n=4 biological replications). * significant at P<0.05 (t-test); ** significant at P<0.05 with Bonferroni correction (continue).

| Metabolite | | Low P/adequate P | | | | RAC875/Wyalkatchem | | | |
| --- | --- | --- | --- | --- | --- | --- | --- | --- | --- |
|  |  | RAC875 | | Wyalkatchem | | P10 | | P30 | |
|  |  | P10/ P30 | sem | P10/ P30 | sem | R/W | sem | R/W | sem |
| Glutamic acid | 0.860 | | 0.140 | 0.840 | 0.080 | 0.901 | 0.140 | 0.879 | 0.127 |
| 2- oxo-glutaric acid | 0.765 | | 0.248 | 0.656 | 0.138 | 0.790 | 0.248 | 0.677 | 0.198 |
| Glyceric acid | 1.156 | | 0.042 | 1.363* | 0.055 | 0.772* | 0.042 | 0.910 | 0.140 |
| Glycolic acid | 0.629* | | 0.099 | 1.108 | 0.161 | 0.654 | 0.099 | 1.152 | 0.117 |
| Iso Citric | 1.016 | | 0.021 | 1.070 | 0.061 | 0.893 | 0.021 | 0.941 | 0.079 |
| Itaconate | 0.378* | | 0.127 | 1.053 | 0.232 | 0.468* | 0.127 | 1.305 | 0.166 |
| Maleate | 0.552 | | 0.095 | 1.181 | 0.121 | 0.519* | 0.095 | 1.111 | 0.170 |
| Malic acid | 1.209 | | 0.055 | 1.287* | 0.081 | 0.700* | 0.055 | 0.745 | 0.149 |
| Malonic acid | 1.123 | | 0.089 | 1.095 | 0.126 | 0.705 | 0.089 | 0.687 | 0.200 |
| Nonaoic acid | 1.083 | | 0.024 | 1.179 | 0.057 | 1.066 | 0.024 | 1.161 | 0.097 |
| Pipecolate | 0.568 | | 0.235 | 0.684* | 0.122 | 0.803 | 0.235 | 0.968 | 0.234 |
| Pyruvic acid | 1.084 | | 0.109 | 0.921 | 0.114 | 1.052 | 0.109 | 0.894 | 0.107 |
| Quinic acid | 1.375 | | 0.117 | 1.181 | 0.130 | 0.760 | 0.117 | 0.652 | 0.260 |
| Saccharic acid | 1.058 | | 0.073 | 0.988 | 0.084 | 1.392* | 0.073 | 1.300 | 0.155 |
| Shikimic acid | 1.436 | | 0.090 | 1.151 | 0.114 | 1.313 | 0.090 | 1.053 | 0.133 |
| Succinic acid | 0.944 | | 0.093 | 0.878 | 0.046 | 0.829 | 0.093 | 0.771 | 0.150 |
| Threonic acid | 1.014 | | 0.048 | 1.018 | 0.060 | 0.683* | 0.048 | 0.686^*^ | 0.134 |
| Sugars, Sugar Alcohol and Sugar Phosphates |  | |  |  |  |  |  |  |  |
| 1-kestose | 2.952* | | 0.175 | 2.049* | 0.142 | 2.026* | 0.175 | 1.406 | 0.269 |
| Arabinose | 0.966 | | 0.136 | 1.178* | 0.017 | 0.919 | 0.136 | 1.121 | 0.108 |
| Arabitol | 0.871 | | 0.037 | 1.272 | 0.296 | 0.811 | 0.037 | 1.184 | 0.389 |
| Beta Gentibiose | 1.445 | | 0.182 | 1.431 | 0.321 | 0.727 | 0.182 | 0.720 | 0.113 |
| Fructose | 0.933 | | 0.117 | 0.993 | 0.052 | 0.848 | 0.117 | 0.903 | 0.186 |
| Fructose-6-phosphate | 1.173 | | 0.161 | 0.530* | 0.147 | 1.334 | 0.161 | 0.603 | 0.176 |
| Fucose | 1.041 | | 0.136 | 1.058 | 0.084 | 1.054 | 0.136 | 1.071 | 0.212 |
| Galactinol | 0.996 | | 0.053 | 0.890 | 0.110 | 1.063 | 0.053 | 0.949 | 0.281 |
| Galactose | 0.900 | | 0.140 | 1.003 | 0.062 | 0.835 | 0.140 | 0.930 | 0.204 |
| Galactosylglycerol | 1.531 | | 0.102 | 2.017* | 0.087 | 0.772 | 0.102 | 1.017 | 0.174 |
| Glucose | 0.889 | | 0.141 | 1.014 | 0.060 | 0.826 | 0.141 | 0.942 | 0.207 |
| Glucose-6-phosphate | 1.003 | | 0.126 | 0.492* | 0.149 | 1.306 | 0.126 | 0.641 | 0.174 |
| Inositol myo | 1.015 | | 0.082 | 1.083 | 0.063 | 0.915 | 0.082 | 0.976 | 0.136 |
| Maltose | 1.300 | | 0.097 | 1.468 | 0.033 | 1.016 | 0.097 | 1.148 | 0.230 |
| Mannitol | 1.330 | | 0.164 | 2.374 | 0.398 | 0.695 | 0.164 | 1.240 | 0.392 |
| Mannose | 1.307 | | 0.150 | 1.283* | 0.039 | 1.108 | 0.150 | 1.087 | 0.189 |
| Raffinose | 2.805* | | 0.159 | 2.002 | 0.133 | 1.897* | 0.159 | 1.354 | 0.252 |

Table S 4. Fold-change in metabolite levels in roots of efficient (RAC875) and inefficient (Wyalkatchem). Comparisons between low P supply and adequate P supply (low P/adequate P); and between RAC875 and Wyalkatchem (RAC875/Wyalkatchem) at each P supply. R: RAC875; W: Wyalkatchem; Low P: 10 mg P kg^-1^ soil (P10), adequate P: 30 mg P kg^-1^ soil (P30); sem: standard error of the mean (n=4 biological replications). * significant at P<0.05 (t-test); ** significant at P<0.05 with Bonferroni correction (continue).

| Metabolite | | Low P/adequate P | | | | RAC875/Wyalkatchem | | | |
| --- | --- | --- | --- | --- | --- | --- | --- | --- | --- |
|  |  | RAC875 | | Wyalkatchem | | P10 | | P30 | |
|  |  | P10/ P30 | sem | P10/ P30 | sem | R/W | sem | R/W | sem |
| Rhamnose | 1.087 | | 0.103 | 1.015 | 0.088 | 1.012 | 0.103 | 0.945 | 0.145 |
| Ribitol | 1.017 | | 0.110 | 0.828* | 0.073 | 1.035 | 0.110 | 0.843 | 0.176 |
| Ribose | 1.080 | | 0.095 | 0.989 | 0.094 | 1.027 | 0.095 | 0.941 | 0.159 |
| Sucrose | 1.343 | | 0.075 | 1.046 | 0.048 | 1.180 | 0.075 | 0.919 | 0.101 |
| Trehelose | 0.946 | | 0.072 | 1.001 | 0.083 | 1.090 | 0.072 | 1.153 | 0.167 |
| Xylitol | 0.972 | | 0.085 | 1.013 | 0.080 | 1.031 | 0.085 | 1.074 | 0.128 |
| Xylose | 1.182 | | 0.042 | 1.268* | 0.047 | 0.927 | 0.042 | 0.995 | 0.144 |
| Others |  | |  |  |  |  |  |  |  |
| Benzoic acid, 4-hydroxy- | 0.589* | | 0.123 | 0.938 | 0.149 | 0.679 | 0.123 | 1.079 | 0.135 |
| Glycerol | 0.886 | | 0.082 | 0.952 | 0.091 | 0.882 | 0.082 | 0.947 | 0.183 |
| Glycerol-3-P | 0.547* | | 0.137 | 0.523* | 0.099 | 0.946 | 0.137 | 0.905 | 0.163 |
| Hexadecanoic acid | 1.185 | | 0.047 | 1.208 | 0.064 | 1.128 | 0.047 | 1.150 | 0.118 |
| Monomethylphosphate | 1.321 | | 0.267 | 0.791 | 0.212 | 1.094 | 0.267 | 0.655 | 0.178 |
| Octadecadienoic acid,9,12-Z,Z | 1.443 | | 0.151 | 1.016 | 0.230 | 1.201 | 0.151 | 0.846 | 0.141 |
| Octadecanoic acid, n- | 1.540 | | 0.129 | 1.151 | 0.120 | 1.474 | 0.129 | 1.101 | 0.124 |
| Octadecatrienoic acid, 9,12,15-Z,Z | 1.489 | | 0.133 | 0.980 | 0.303 | 1.014 | 0.133 | 0.668 | 0.093 |
| Phosphoric acid | 0.360** | | 0.056 | 0.358** | 0.072 | 0.727* | 0.056 | 0.724* | 0.098 |
| Threonic acid-1,4-lactone | 0.774 | | 0.050 | 0.834 | 0.082 | 0.583* | 0.050 | 0.629* | 0.156 |
| Urea | 1.460 | | 0.134 | 2.613* | 0.117 | 0.825 | 0.134 | 1.476 | 0.213 |

Table S 5. Effect of P supply on succinic, maleic, oxalic and citric acids of two wheat genotypes RAC875 and Wyalkatchem at 41 days after sowing (DAS). N=5 biological replications.

| Genotype | P supply (mg P kg^-1^ soil) | Organic acid exudation (µg 15 mL^-1^) | | | |
| --- | --- | --- | --- | --- | --- |
|  |  | Citrate | Maleate | Oxalic | Succinate |
| RAC875 | 5 | 1.80 | 0.45 | 0.45 | 0.94 |
|  | 10 | 2.40 | 0.32 | 0.30 | 0.86 |
|  | 30 | 1.57 | 0.16 | 0.19 | 0.61 |
| Wyalkatchem | 5 | 2.22 | 0.20 | 037 | 0.84 |
|  | 10 | 3.28 | 0.25 | 0.62 | 1.02 |
|  | 30 | 3.00 | 0.18 | 0.46 | 0.88 |
| F prob |  |  |  |  |  |
| Genotype (G) |  | P=0.031 | P=0.036 | P=0.028 | P=0.337 |
| P treatment (P) |  | P=0.242 | P=0.025 | P=0.285 | P=0.038 |
| G × P |  | P=0.558 | P=0.053 | P=0.074 | P=0.347 |
| F test |  |  |  |  |  |
| df_G_ |  | 1 | 1 | 1 | 1 |
| df_P_ |  | 2 | 2 | 2 | 2 |
| df_e_ |  | 16 | 16 | 16 | 16 |

Table S 6. Effect of P supply on succinic, maleic, oxalic and citric acid concentrations of each wheat genotype, RAC875 and Wyalkatchem at 41 days after sowing (DAS) (One-way Anova analysis). N=5 biological replications.

| Genotype | P supply (mg P kg^-1^ soil) | Organic acid exudation (µg 15 mL^-1^) |  |  |  |
| --- | --- | --- | --- | --- | --- |
|  |  | Citrate | Maleate | Oxalic | Succinate |
| RAC875 | 5 | 1.80 | 0.45 | 0.45 | 0.94 |
|  | 10 | 2.40 | 0.32 | 0.30 | 0.86 |
|  | 30 | 1.57 | 0.16 | 0.19 | 0.61 |
| *P* value |  | 0.474 | 0.032 | 0.076 | 0.023 |
|  |  |  |  |  |  |
| Wyalkatchem | 5 | 2.22 | 0.20 | 0.37 | 0.84 |
|  | 10 | 3.28 | 0.25 | 0.62 | 1.02 |
|  | 30 | 3.00 | 0.18 | 0.45 | 0.88 |
| *P* value |  | 0.474 | 0.569 | 0.212 | 0.290 |

Table S 7. Comparisons for succinic, maleic, oxalic and citric acids between P supply of each wheat genotype (one-way Anova, Tukey test)

| Organic acid | Genotype | P supply (mg P kg^-1^ soil) |  | Mean difference | SE | Sig. |
| --- | --- | --- | --- | --- | --- | --- |
| Citrate | RAC875 | 5 | 10 | -.5992993 | .6999864 | .683 |
|  | RAC875 | 5 | 30 | .2347043 | .6547773 | .932 |
|  | RAC875 | 10 | 30 | .8340036 | .6547773 | .452 |
|  | Wyalkatchem | 5 | 10 | -1.0613014 | .6483639 | .280 |
|  | Wyalkatchem | 5 | 30 | -.7817378 | .6483639 | .479 |
|  | Wyalkatchem | 10 | 30 | .2795636 | .6483639 | .904 |
| Maleate | RAC875 | 5 | 10 | .1354615 | .0918370 | .358 |
|  | RAC875 | 5 | 30 | .2926740^*^ | .0859056 | .027 |
|  | RAC875 | 10 | 30 | .1572126 | .0859056 | .229 |
|  | Wyalkatchem | 5 | 10 | -.0520620 | .0645141 | .708 |
|  | Wyalkatchem | 5 | 30 | .0153483 | .0645141 | .969 |
|  | Wyalkatchem | 10 | 30 | .0674103 | .0645141 | .569 |
| Oxalic | RAC875 | 5 | 10 | .1501093 | .1000124 | .347 |
|  | RAC875 | 5 | 30 | .2584513^*^ | .0935531 | .064 |
|  | RAC875 | 10 | 30 | -.1501093 | .1000124 | .347 |
|  | Wyalkatchem | 5 | 10 | -.2554940 | .1350086 | .196 |
|  | Wyalkatchem | 5 | 30 | -.0858486 | .1350086 | .805 |
|  | Wyalkatchem | 10 | 30 | .1696454 | .1350086 | .452 |
| Succinate | RAC875 | 5 | 10 | .0765738 | .1024566 | .745 |
|  | RAC875 | 5 | 30 | .3292789^*^ | .0958394 | .026 |
|  | RAC875 | 10 | 30 | .2527052 | .0958394 | .076 |
|  | Wyalkatchem | 5 | 10 | -.1781284 | .1539468 | .506 |
|  | Wyalkatchem | 5 | 30 | .0749689 | .1539468 | .879 |
|  | Wyalkatchem | 10 | 30 | .2530973 | .1539468 | .278 |

^*^Significant difference
